# The Possible Role of PM_2.5_ Chronic Exposure on 5-Year Survival in Patients with Left Ventricular Dysfunction Following Coronary Artery Bypass Grafting

**DOI:** 10.3390/toxics12100697

**Published:** 2024-09-26

**Authors:** Tomasz Urbanowicz, Krzysztof Skotak, Anna Olasińska-Wiśniewska, Krzysztof J Filipiak, Aleksandra Płachta-Krasińska, Jakub Piecek, Beata Krasińska, Zbigniew Krasiński, Andrzej Tykarski, Marek Jemielity

**Affiliations:** 1 Cardiac Surgery and Transplantology Department, Poznan University of Medical Sciences, 61-701 Poznan, Poland; 2 Institute of Environmental Protection–National Research Institute, 02-170 Warsaw, Poland; 3 Institute of Clinical Science, Maria Sklodowska-Curie Medical Academy, 00-136 Warsaw, Poland; 4 Department of Hypertensiology, Angiology and Internal Medicine, Poznan University of Medical Sciences, 61-701 Poznan, Poland; 5 Department of Ophthalmology, Poznan University of Medical Sciences, 61-701 Poznan, Poland; 6 Student Research Group, Medical Faculty, Poznan University of Medical Sciences, 61-107 Poznan, Poland; 7 Department of Vascular, Endovascular Surgery, Angiology and Phlebology, Poznan University of Medical Science, 61-701 Poznań, Poland

**Keywords:** OPCAB, CAD, air pollution, PM_2.5_, LVEF

## Abstract

Background: The survival benefit of surgical revascularization in multivessel coronary artery disease is well understood, though it can be modified by left ventricular dysfunction. Chronic exposure to air pollutants has gained more attention recently as a possible non-traditional morbidity and mortality cardiovascular risk factor. This study identified possible 5-year mortality risk factors related to postoperative left ventricular performance, including air pollutants. Patients: There were 283 patients (244 (86%) males) with a median age of 65 (60–70) years enrolled in the retrospective analysis. All patients were referred for off-pump coronary artery revascularization due to chronic coronary syndrome that presented as a multivessel coronary artery disease. They were divided into three groups depending on the postoperative course of left ventricular fraction (LVEF 50% or more (169 patients), LVEF between 41 and 49% (61 patients), and LVEF 40% or less (53 patients)). Results: The overall survival rate was 84% (237 patients) in a median follow-up time of 5.3 (4.8–6.1) years. The median (Q1–Q3) chronic air pollution exposures for the analyzed group were 19.3 (16.9–22.4) μg/m^3^ for fine particles such as PM_2.5_, 25.8 (22.5–29.4) μg/m^3^ for coarse particles such as PM_10_, and 12.2 (9.7–14.9) μg/m^3^ for nitric dioxide (NO_2_). The mortality in the first group (LVEF at least 50%) was 23 (13.6%), in the second group (LVEF 41–49%) was 9 (15%), and in the third group (LVEF 40% or less) was 14 (26%). The multivariable regression analysis for the five-year mortality risk in the first group revealed the predictive value of dyslipidemia (HR: 3.254, 95% CI: 1.008–10.511, *p* = 0.049). The multivariable regression analysis for five-year mortality risk in the second group revealed the predictive value of dyslipidemia (HR: 3.391, 95% CI: 1.001–11.874, *p* = 0.050) and PM_2.5_ (HR: 1.327, 95% CI: 1.085–1.625, *p* = 0.006). In the third group (severely decreased LVEF), chronic PM_2.5_ exposure was found to be significant (HR: 1.518, 95% CI: 1.50–2.195, *p* = 0.026) for 5-year mortality prediction. Conclusions: Traditional risk factors, such as dyslipidemia, are pivotal in the 5-year mortality risk following surgical revascularization. Chronic exposure to ambient air pollutants such as PM_2.5_ may be an additional risk factor in patients with left ventricular dysfunction.

## 1. Introduction

Coronary artery atherosclerotic disease is characterized by an increased mortality risk. Non-traditional risk factors such as air pollution have gained more attention due to constant climate change [1,2]. Our previous analysis revealed the possible relationship between coronary artery disease progression, air pollution [3], and ambient temperature [4].

The current assessment of ischemic disease advancement involves anatomical and functional evaluation to optimize symptom reduction and address major adverse cardiovascular event threats [5]. The percutaneous and surgical interventions present satisfactory results and indicate a personalized approach [6]. In the meta-analysis at the 5-year follow-up by Formica et al. [7], higher incidences of all-cause mortality, myocardial infarction, and repeat revascularization were revealed among patients with multivessel coronary disease or left main disease treated with percutaneous interventions.

The two surgical techniques, off-pump and on-pump surgery, did not reveal significant long-term outcome differences in the randomized trial of Quin et al. [8]. Though still limited in overall application number, the off-pump surgical technique presents satisfactory results, especially in high-risk patients [8,9,10]. In the results of the recently published SYNTASES trial [7], 10-year mortality adjusted for significant confounders was significantly lower following on-pump surgical revascularization than with off-pump and percutaneous approaches.

Although the survival benefit of surgical revascularization in multivessel coronary artery disease is well understood, it can be modified by left ventricular dysfunction. Previous analysis pointed out the survival benefit of surgical over percutaneous therapies in multivessel disease in patients with left ventricular dysfunction [11]. Even asymptomatic mild left ventricular impaired function limits the prognosis and may progress to more advanced stages [12]. Heart dysfunction induces inflammatory activation related to mitochondrial dysfunction [13]. The presented phenomenon is characterized by impaired energy production, oxidative stress, and disrupted calcium homeostasis. Airborne fine particles are one of the strong external stimuli for inflammatory activation [14].

Accurately managing traditional coronary artery disease risk factors is essential for long-term results optimization. In their meta-analysis, Bond et al. [8] presented the relationship between ambient air pollution exposure and increased risk for all-cause cardiovascular morbidity and morbidity. Our previous studies revealed an increased risk for coronary disease progression related to air pollutants [15].

This study aimed to identify possible 5-year mortality risk factors, including air pollutants related to postoperative left ventricular performance. The mortality risk assessment was performed based on demographical and clinical characteristics, including non-traditional cardiovascular elements such as environmental factors.

## 2. Materials and Methods

There were 283 consecutive patients (244 (86%) males) with a median age of 65 (60–70) years enrolled in the retrospective analysis. All patients were referred for off-pump coronary artery revascularization due to chronic coronary syndrome, which presented as a multivessel disease. Co-morbidities that characterized the patients included arterial hypertension (222 (78%)), dyslipidemia (149 (53%)), and diabetes mellitus (111 (39%)). They were divided into three groups according to the current classification of heart failure based on the postoperative course of left ventricular ejection fraction (LVEF 50% or more (169 patients), an LVEF between 41–49% (61 patients), and an LVEF 40% or less (53 patients), as presented in Table 1.

### 2.1. Air Pollution Exposure Methodology

Three health-relevant air pollutants were considered for our study: particulate matter with a diameter of 10 microns or less (PM_10_), particulate matter with a diameter of 2.5 microns or less (PM_2.5_), and nitrogen dioxide (NO_2_). 

The level of individual patients’ exposure was assessed using spatial distributions of air concentration fields across Poland, as provided by the Chief Inspectorate of Environmental Protection. Maps of air pollutants PM_10_, PM_2.5_, and NO_2_ were derived from the results of the National Air Quality Modelling (NAQM) system, elaborated by the Institute of Environmental Protection–National Research Institute in Poland (IEP-NRI), in line with the Environmental Protection Act in Poland (Art 66, paragraph 6). The NAQM base consists of two components: (1) high-resolution bottom-up emission inventory maps of air pollutants stored in the Central Emission Database [16] and (2) air concentration maps elaborated using the GEM-AQ model, which operates in the Copernicus Atmosphere Monitoring Service—Regional Production (CAMS2_40) [17].

### 2.2. Statistical Analysis

The normality of the distribution of variables was tested with the Shapiro–Wilk test. The t-test, Cochran–Cox test, Mann–Whitney tests, and Fisher’s exact test were used where applicable to compare the variables between groups. Multivariable Cox regression was performed to analyze the predictors of long-term mortality. Demographic (age, sex, body mass index (BMI)), clinical (arterial hypertension, diabetes mellitus, hypercholesterolemia, peripheral artery disease, surgical details), laboratory (troponin, creatinine, uric acid), and air pollution (PM_2.5_, PM_10_, NO_2_) data were evaluated. Statistical analysis was performed using Statistica 13 by TIBCO. *p* < 0.05 was considered statistically significant. 

### 2.3. Bioethics Committee

Informed consent was obtained from all participants. This study was conducted in accordance with the Declaration of Helsinki and approved by the Institutional Review Board (or Ethics Committee) of Poznan University of Medical Sciences, Poznan, Poland (protocol code 55/20 from 16 January 2020), for studies involving humans.

## 3. Results

The overall survival rate was reported to be 84% (237 patients) in a median follow-up time of 5.3 (4.8–6.1) years. There were no perioperative deaths and no major adverse coronary events reported in the analyzed group. All patients were operated on through median sternotomy in the off-pump technique. The mean graft number was 2.3 (0.7), and the median hospitalization time was 12 (9–14) days, as presented in Table 2. 

Postoperative exposure to ambient air pollutants was calculated individually for each patient. The median (Q1–Q3) chronic air pollution exposures for the analyzed group were 19.3 (16.9–22.4) μg/m^3^ for fine particles such as PM_2.5_, 25.8 (22.5–29.4) μg/m^3^ for coarse particles such as PM_10_, and 12.2 (9.7–14.9) μg/m^3^ for nitric dioxide (NO2). The mortality rates in groups were as follows: in the first group (LVEF at least 50%) 23 (13.6%), in the second group (LVEF 41–49%) 9 (15%), and in the third group (LVEF 40% or less) 14 (26%) patients died. The detailed follow-up information is presented in Table 3.

### 3.1. Logistic Regression Analysis

The multivariable Cox regression analysis for predicting 5-year all-cause mortality risk factors was performed separately for each group. This study included ambient air pollutant exposure in places of habitation, including fine particles such as PM_2.5_ and coarse particles such as PM_10_ and NO_2_.

### 3.2. Group 1

The univariable and multivariable Cox analysis of 5-year mortality risk in the first group (LVEF 50% or less) revealed the predictive value of dyslipidemia (HR: 3.254, 95% CI: 1.008–10.511, *p* = 0.049), presented in Table 4.

### 3.3. Group 2

The multivariable stepwise regression analysis for 5-year mortality risk in the second group (LVEF 40–49%) revealed the predictive value of dyslipidemia (OR: 3.391, 95% CI: 1.001–11.874, *p* = 0.050) and PM_2.5_ (OR: 1.327, 95% CI: 1.085–1.625, *p* = 0.006) as presented in Table 5.

### 3.4. Group 3

The multivariable analysis stepwise regression analysis for 5-year mortality risk in the third group (LVEF 40% or less) revealed the predictive value of chronic PM_2.5_ exposure (OR: 1.518, 95% CI: 1.50–2.195, *p* = 0.026), as shown in Table 6.

## 4. Discussion

Our analysis points out the significance of non-traditional mortality risk factors such as air pollution alongside dyslipidemia in coronary disease patients who underwent surgical revascularization. Eugene Braunwald has already presented the influence of environmental factors, including ambient pollution, on increased mortality risk [18].

We confirmed the prognostic value of dyslipidemia on patients’ survival following coronary artery revascularization. According to epidemiological studies, lipid-lowering therapy may decrease mortality risk in the current population, as coronary heart disease is the single leading cause of over 40% of CVD deaths [19]. Atherosclerosis is an age-related disorder representing the complex mechanisms leading to lipid-rich lesion formation in the circulatory system. The intricate balance between endothelium-derived relaxing factors, such as nitric oxide and prostacyclins, and contracting factors, such as superoxide anion and endothelin-1, is disturbed in atherosclerotic lesion formation, especially in dyslipidemic patients [20]. The impaired endothelial hemostasis is a critical contributor to aging and chronic cardiometabolic disorders. The mechanism of plaque development relies on inflammatory activation and involves various types of cells, including macrophages, endothelial, vascular smooth muscle cells, and endothelial progenitor cells that are induced. Recent studies highlight another process that may play a significant role in the mentioned process and that is stimulated by dyslipidemia, named cellular senescence [21]. Prasad, in his review [22], pointed out the significance of modifiable risk factor controls, like arterial hypertension, dyslipidemia, diabetes mellitus, hypertension, obesity, and chronic renal disease for primary, secondary, and even tertiary preventive care. The low-density lipoprotein concentration is considered a primary target in cardiovascular patients [23]. Our previous analysis revealed the protective role of LDL lowering in perioperative myocardial injury in coronary revascularization [24]. The study by Lim et al. presented the association between exposure to elevated LDL and non-HDL levels and increased postoperative mortality [25]. Our analysis highlights the significance of dyslipidemia’s presence, despite statin therapy, on 5-year survival in surgically treated patients with multivessel coronary disease.

The exploration of the possible role of air pollution in long-term survival, especially in patients presenting with decreased ejection fraction, is the novelty of our analysis. The environmental factors may be prognostic factors of worse outcomes in certain groups of patients following surgical coronary revascularization. The decreased ejection fraction following the surgical revascularization signifies the heart failure-related inflammatory activation. Regardless of the underlying etiology, heart dysfunction induces cytokines and chemokines that modulate the phenotype and function of all myocardial cells, inflammatory activation in macrophages, and microvascular dysfunction [26]. Systemic inflammatory markers, presented as possible late mortality risk predictors [27] related to left ventricular dysfunction, were reported to decrease in coordination with myocardial improvement [28]. In the CANTOS trial, the use of anti-inflammatory therapies, following lipid-lowering strategies, led to significantly lower MACE risks [29].

Air pollutants induce inflammatory activation [30]. Fine particulate matter below 2.5 μm in diameter (PM_2.5_) mainly arises from fossil fuel combustion during power generation, transportation, and industrial processes and has been identified as the main hazardous constituent [31]. PM_2.5_ can cross the alveolar–capillary barrier, reach other body organs, and activate tissue-resident immune cells, inducing oxidative stress, triggering inflammatory reactions, and stimulating the autonomic nervous system. In experimental studies, the properties of PM_2.5_ in vascular cell penetration and its direct toxic effects were investigated [32]. PM_2.5_ can alter mitochondrial DNA and gene expression at the cellular level, resulting in dysfunction that may lead to cell death [33]. The relationship between ambient PM_2.5_ and increased serum cardiac biomarkers and inflammatory and oxidate stress indices is postulated [34,35]. Chronic exposure to PM_2.5_ is currently regarded as a subclinical marker of atherosclerosis and CV-related increased mortality [36]. This is the main novelty of our analysis, namely, pointing out the significance of environmental factors influencing predisposed patients in whom the inflammatory processes have already been activated. Our results bring a new perspective to ambient pollution exposure in the cardiovascular population, suggesting that the presented effect can be more pronounced in predisposed patients.

Epidemiological studies have already presented the association between PM_2.5_ exposure and increased mortality risk [37]. The unique characteristic of our analysis is the personalized approach. The exposure to ambient air pollutants was separately calculated for each patient, indicating its influence on human organisms. We focused on patient-calculated chronic exposure to ambient pollution, suggesting its role in overall mortality. However, previous studies highlighted the significance of acute and chronic PM_2.5_ changes in increased mortality risk [38].

Study limitation: The study was a single-center analysis performed on patients presenting with chronic coronary syndrome who were diagnosed with multivessel coronary disease. However, all patients underwent off-pump surgical revascularization in a high-volume center well experienced in the mentioned technique. The second limitation is the fact that study results are based on all-cause mortality results.

## 5. Conclusions

The traditional risk factors, such as arterial hypertension, play a pivotal role in the 5-year mortality risk following surgical revascularization. Chronic exposure to ambient air pollutants such as PM_2.5_ may be regarded as an additional risk factor in patients after surgical revascularization with left ventricular dysfunction.

## Figures and Tables

**Table 1:** Groups’ demographical and clinical characteristics.

Parameters	Group 1	Group 2	Group 3	*p*	*p*	*p*
	LVEF ≥ 50%	LVEF 41–49%	LVEF ≤ 40%	Group 1 vs. Group 2	Group 1 vs. Group 3	Group 2 vs. Group 3
	n = 169	n = 61	n = 53			
Demographical						
Age (years) (median (Q1–Q3)	64 (60–72)	64 (58–69)	64 (59–68)	0.322	0.186	0.795
Sex (male (%))	142 (84)	52 (85)	49 (92)	0.627	0.124	0.324
BMI (median (Q1–Q3)	28.4 (26.6–30.9)	28.4 (26.3–31.0)	28.7 (26.6–31.5)	0.624	0.74	0.532
Co-morbidities						
Arterial hypertension (n, %)	128 (76)	49 (80)	45 (85)	0.349	0	0.302
Dyslipidemia (n, %)	89 (53)	30 (49)	30 (57)	0.724	0.457	0.639
Diabetes mellitus (n, %)	57 (34)	21 (34)	19 (36)	0.859	0.778	0.928
PAD (n, %)	18 (11)	5 (8)	4 (8)	0.61	0.512	0.883
CAD diagnosis:						
Left main disease (n, %)	51 (30)	19 (31)	14 (26)	0.873	0.73	0.68
Two-vessel disease (n, %)	49 (29)	18 (30)	14 26)	1	0.862	0.835
Three-vessel disease (n, %)	69 (41)	24 (39)	25 (47)	0.88	0.43	0.451

Abbreviations: BMI—body mass index, CAD—coronary artery disease, LVEF—left ventricular ejection fraction, n—number.

**Table 2:** Perioperative characteristics.

Parameters	Group 1	Group 2	Group 3	*p*	*p*	*p*
	LVEF ≥ 50%	LVEF 41–49%	LVEF ≤ 40%	Group 1 vs. Group 2	Group 1 vs. Group 3	Group 2 vs. Group 3
	n = 169	n = 61	n = 53			
Preoperative laboratory results:						
WBC (×10^9^/L) (median (Q1–Q3))	7.70 (6.47–8.93)	7.84 (6.41–8.83)	7.44 (6.59–8.51)	0.612	0.738	0.716
Hb (mmol/L) (median (Q1–Q3))	8.8 (8.2–9.3)	8.7 (8.2–9.15)	8.90 (8.40–9.30)	0.972	0.699	0.416
Plt (×10^9^/L) (median (Q1–Q3))	219 (187–259)	225 (188–262)	222 (182–263)	0.896	0.961	0.863
Creatinine (μmol/L) (median (Q1–Q3))	98 (79–114)	93 (74–108)	95 (89–107)	0.578	0.685	0.893
CRP (mg/L) (median (Q1–Q3))	6 (5–8)	6 (3–8)	6 (5–8)	0.64	0.934	0.756
Preoperative echocardiography:						
LVED (mm) (median (Q1–Q3))	50 (44–55)	57 (53–60)	61 (57–64)	<0.001	<0.001	<0.001
LVEF (%) (median (Q1–Q3))	53 (50–57)	40 (38–43)	31 (27–34)	<0.001	<0.001	<0.001
Off-pump surgery:						
Skin-to-skin time (min) (median (Q1–Q3))	132 (119–167)	139 (121–170)	161 (120–182)	0.289	0.116	0.189
Number of grafts (n, mean (SD))	2.2 (0.8)	2.4 (0.7)	3.0 (0.8)	0.148	0.047	0.563
Troponin max (ng/mL) (median (Q1–Q3))	1.698 (0.789–4.334)	1.47 (0.603–3.416)	2.37 (0.942–4.125)	0.452	0.453	0.187
Overall hospitalization: (days) (mean (SD))	10 (2)	11 (3)	14 (3)	0.608	0.043	0.278
Complications:						
Bleeding (n, (%))	2 (1)	1 (2)	1 (2)	1	1	0.561
Wound infection (n, (%))	3 (2)	2 (3)	1 (2)	0.61	1	1

Abbreviations: CRP—C-reactive protein, Hb—hemoglobin, LVED—left ventricular end-diastolic diameter, LVEF—left ventricular ejection fraction, n—number, Plt—platelet count, Q—quartile, SD—standard deviation, WBC—white blood cells count.

**Table 3:** Patients’ characteristics in follow-up.

Parameters	Group 1	Group 2	Group 3	*p*	*p*	*p*
	LVEF ≥ 50%	LVEF 41–49%	LVEF ≤ 40%	Group 1 vs. Group 2	Group 1 vs. Group 3	Group 2 vs. Group 3
	n = 169	n = 61	n = 53			
Mean follow-up time (years) (mean (SD)	5.3 (1.1)	5.5 (1.1)	5.4 (1.1)	0.919	0.814	0.818
Follow–up laboratory results:						
WBC (×10^9^/L) (median (Q1–Q3))	8.32 (7.04–9.73)	8.6 (6.96–10.31)	8.91 (7.68–10.49)	0.797	0.152	0.535
Hb (mmol/L) (median (Q1–Q3))	7.0 (6.6–7.4)	6.8 (6.5–7.45)	6.9 (6.5–7.5)	0.915	0.767	0.709
Plt (×10^9^/L) (median (Q1–Q3))	264 (211–322)	258 (220–303)	272 (222–354)	0.988	0.509	0.502
Creatinine (μmol/L) (median (Q1–Q3))	92 (79–104)	94 (75–105)	93 (81.5–100.6)	0.589	0.847	0.892
Uric acid (μmol/L) (median (Q1–Q3))	5.72 (4.85–6.99)	5.94 (5.01–6.63)	6.00 (4.98–7.64)	0.961	0.346	0.946
Hb1Ac (%) (median (Q1–Q3))	6.4 (6.0–6.9)	6.5 (6.1–7.1)	6.4 (6.0–7.0)	0.928	0.879	0.945
Lipidogram:						
Total cholesterol (mmol/L) (median (Q1–Q3))	4.0 (3.3–4.7)	3.7 (3.1–4.2)	3.8 (3.5–4.2)	0.131	0.212	0.441
LDL (mmol/L) (median (Q1–Q3))	2.2 (1.6–2.9)	1.7 (1.3–2.4)	2.1 (1.6–2.3)	0.034	0.145	0.123
HDL (mmol/L) (median (Q1–Q3))	1.2 (0.9–1.5)	1.0 (0.9–1.3)	1.1 (1.0–1.2)	0.11	0.48	0.423
TG (mmol/L) (median (Q1–Q3))	1.4 (1.1–1.8)	1.5 (1.0–1.9)	1.5 (1.0–1.5)	0.324	0.052	0.365
Follow-up echocardiography						
LVED (mm) (median) (Q1–Q3)	48 (42–52)	55 (51–58)	57 (53–61)	<0.001	<0.001	<0.001
LVEF (%) (median (Q1–Q3)	55 (50–60)	44 (41–47)	33 (30–37)	<0.001	<0.001	<0.001
Postoperative pharmacotherapy:						
B-blockers (n (%))	169 (100)	61 (100)	53 (100)	1	1	1
ACE-I (n (%))	151 (89)	56 (92)	21 (40)	1	<0.001	<0.001
ARNI (n (%))	14 (8)	3 (5)	32 (60)	0.768	<0.001	<0.001
Diuretics (n (%))	29 (17)	17 (28)	34 (64)	0.092	<0.001	<0.001
SGLT2 inhibitors (n (%))	2 (1)	3 (5)	15 (28)	0.09	<0.001	0.004
Statins (n (%))	164 (97)	61 (100)	53 (100)	0.566	1	1
MRA (n (%))	3 (18)	5 (8)	45 (85)	0.034	<0.001	<0.001
ASA (n (%))	169 (100)	61 (100)	53 (100)	1	1	1
Insulin (n (%))	31 (18)	6 (10)	10 (19)	0.218	0.838	0.187
Metformin (n (%))	26 (15)	15 (25)	43 (81)	0.12	<0.001	<0.001
Ambient air pollution						
PM_2.5_ (μg/m^3^) (median (Q1–Q3)	18.9 (16.9–22.4)	20.6 (17.8–23.0)	18.9 (15.4–21.8)	0.152	0.614	0.108
PM_10_ (μg/m^3^) (median (Q1–Q3)	25.3 (22.4–29.6)	26.7 (23.9–29.7)	25.0 (21.2–28.3)	0.237	0.37	0.053
NO_2_ (μg/m^3^) (median (Q1–Q3)	12.2 (9.99–15.62)	13.0 (10.1–15.1)	11.2 (9.3–14.5)	0.709	0.217	0.145
Five-year overall mortality (n, %)	23 (14)	9 (15)	14 (26)	0.831	0.036	0.161

Abbreviations: ACE-I—angiotensin-converting enzyme—inhibitor, ARNI—angiotensin receptor neprilysin inhibitors, ASA—aspirin, BMI—body mass index, Hb—hemoglobin, Hb1Ac—glycemic hemoglobin, HDL—high-density lipoprotein, LDL—low-density lipoprotein, LVED—left ventricular end-diastolic diameter, LVEF—left ventricular ejection fraction, MRA—mineralocorticoid receptor antagonist, n—number, NO2—nitric dioxide, PM_2.5_—air pollution particle matter 2.5 μm or less, PAD—peripheral artery disease, PM_10_—air pollution particle matter 10 μm or less, Plt—platelets, SD—standard deviation, SGLT2—sodium–glucose cotransporter-2, TG—triglycerides, Q—quartile, WBC—white blood cell count.

**Table 4:** Univariable and multivariable analysis for 5-year mortality prediction in patients operated on due to multivessel artery disease presenting in postoperative course normal left ventricular ejection fraction (LVEF >50%).

Parameters	Univariable			Multivariable		
	HR	95% CI	*p*	HR	95% CI	*p*
Demographical:						
Age	0.976	0.828–1.151	0.775			
Sex (male)	0.755	0.135–2.606	0.49			
BMI	0.976	0.828–1.151	0.755			
Clinical:						
Arterial hypertension	2.411	0.465–12.512	0.295			
Diabetes mellitus	2.402	0.702–8.218	0.163			
Hypercholesterolemia	4.246	1.152–15.646	0.03	3.254	1.008–10.511	0.049
PAD	1.145	1.044–3.871	0.437			
Perioperative:						
Number of grafts (2)	1.478	0.288–7.574	0.64			
Number of grafts (3)	1.579	0.309–8.078	0.583			
Arterial revascularization	0.91	0.567–1.245	0.592			
Troponin max	0.903	0.770–1.059	0.21			
Postoperative:						
Creatinine	0.995	0.970–1.022	0.726			
Uric acid	0.889	0.616–1.282	0.528			
Air pollution exposure:						
PM_2.5_	0.979	0.688–1.392	0.906			
PM_10_	0.955	0.723–1.370	0.977			
NO_2_	1.012	0.871–1.175	0.879			

Abbreviations: BMI—body mass index, HR—hazard ratio, NO2—nitric dioxide, PM_2.5_—air pollution particle matter 2.5 μm or less, PAD—peripheral artery disease, PM_10_—air pollution particle matter 10 μm or less.

**Table 5:** Univariable and multivariable analysis for 5-year mortality prediction in patients operated on due to multivessel artery disease presenting in postoperative course reduced left ventricular ejection fraction (LVEF 40–49%).

Parameter	Univariable			Multivariable		
	HR	95% CI	*p*	HR	95% CI	*p*
Demographical:						
Age	0.82	0.521–1.291	0.39			
Sex (male)	0.047	0.01–4.086	0.18			
BMI	1.194	0.743–1.919	0.463			
Clinical:						
Arterial hypertension	4.366	0.280–10.672	0.196			
Diabetes mellitus	1.934	0.124–30.031	0.638			
Hypercholesterolemia	6.767	0.859–83.861	0.156	3.391	1.001–11.874	0.05
PAD	1.04	0.103–5.764	0.241			
Perioperative:						
Number of grafts (2)	0.053	0.001–33.6700	0.226			
Number of grafts (3)	0.062	0.002–43.703	0.227			
Arterial revascularization	0.902	0.567– 1.674	0.997			
Troponin max	1.015	0.805–1.278	0.902			
Postoperative:						
Creatinine	0.997	0.943–1.054	0.913			
Uric acid	1.155	0.282–4.726	0.841			
Air pollution exposure:						
PM_2.5_	2.084	0.849–5.114	0.109	1.327	1.085–1.625	0.006
PM_10_	1.009	0.122–1.250	0.113			
NO_2_	1.429	0.829–2.464	0.199			

Abbreviations: BMI—body mass index, HR—hazard ratio, NO2—nitric dioxide, PM_2.5_—air pollution particle matter 2.5 μm or less, PAD—peripheral artery disease, PM_10_—air pollution particle matter 10 μm or less.

**Table 6:** Univariable and multivariable analysis for 5-year mortality prediction in patients operated on due to multivessel artery disease presenting in postoperative course significantly reduced left ventricular ejection fraction (LVEF < 40%).

Parameter	Univariable			Multivariable		
	HR	95% CI	*p*	HR	95% CI	*p*
Demographical:						
Age	1.032	0.868–1.228	0.719			
Sex (male)	4.061	0.681–10.603	0.996			
BMI	1.035	0.784–1.367	0.807			
Clinical:						
Arterial hypertension	1.374	0.477–21.139	0.633			
Diabetes mellitus	1.856	0.228–15.096	0.563			
Hypercholesterolemia	2.397	0.327–24.812	0.142			
PAD	1.496	0.484–11.671	0.401			
Perioperative:						
Number of grafts (2)	1.478	0.961–1.029	0.743			
Number of grafts (3)	1.579	0.309–8.078	0.583			
Arterial revascularization	0.91	0.567–1.245	0.592			
Troponin max	1.062	0.998–1.158	0.092			
Postoperative:						
Creatinine	0.994	0.970–1.022	0.726			
Uric acid	0.889	0.616–1.282	0.528			
Air pollution exposure:						
PM_2.5_	1.311	0.588–2.923	0.509	1.518	1.050–2.195	0.026
PM_10_	1.322	0.547–3.193	0.535			
NO_2_	0.644	0.306–1.355	0.247			

Abbreviations: BMI—body mass index, HR—hazard ratio, NO2—nitric dioxide, PM_2.5_—air pollution particle matter 2.5 μm or less, PAD—peripheral artery disease, PM_10_—air pollution particle matter 10 μm or less.

## Data Availability

Data supporting the reported results can be obtained by contacting the e-mail address of the correspondence author after presenting the justifiable reasons. Data is not available as a supplemental material due to privacy restrictions.
